# Protective Effects of the mTOR Inhibitor Everolimus on Cytoskeletal Injury in Human Podocytes Are Mediated by RhoA Signaling

**DOI:** 10.1371/journal.pone.0055980

**Published:** 2013-02-13

**Authors:** Stefanie Jeruschke, Anja Katrin Büscher, Jun Oh, Moin Ahson Saleem, Peter Friedrich Hoyer, Stefanie Weber, Perihan Nalbant

**Affiliations:** 1 Pediatric Nephrology, Pediatrics II, University of Duisburg-Essen, Essen, Germany; 2 Children’s Renal Unit, Bristol Royal Hospital for Children, University of Bristol, Bristol, United Kingdom; 3 Department of Pediatric Nephrology, Children’s Hospital, University Medical Center, Hamburg, Germany; 4 Center for Medical Biotechnology, Molecular Cell Biology, University of Duisburg-Essen, Essen, Germany; Tohoku University, Japan

## Abstract

Podocytes are highly differentiated kidney cells playing an important role in maintaining the glomerular filtration barrier. Particularly, the integrity of the actin cytoskeleton is crucial as cytoskeletal damage associated with foot process effacement and loss of slit diaphragms constitutes a major aspect of proteinuria. Previously, the mammalian target of rapamycin (mTOR) was linked to actin regulation and aberrant activity of the kinase was associated with renal disease. In this study, actin-related effects of mTOR inhibition by the immunosuppressant everolimus (EV) were investigated in human podocytes using an in vitro model of puromycin aminonucleoside (PAN) induced proteinuria. EV substantially recovered aberrant podocyte behavior by re-establishing a stationary phenotype with decreased migration efficiency, enhanced cell adhesion and recovery of actin stress fibers. Biochemical studies revealed substantial increase in the activity of RhoA and the effector pathway Rho-associated protein kinase (ROCK) and myosin light chain (MLC) by EV, all known regulators of stress fiber generation. Taken together, we show for the first time cytoskeleton stabilizing effects of the mTOR inhibitor EV and establish RhoA signaling as a key mediator in this process.

## Introduction

Differentiated podocytes constitute a major component of the glomerular filtration barrier of the kidney by forming prominent and interdigitating foot processes and the interjacent slit diaphragms, highly specialized intercellular junctions. The integrity of this complex cellular morphology is crucial for proper glomerular function. Severe disorders of the kidney that present with proteinuria are associated with marked foot process effacement, a flattening and broadening of the processes with loss of slit diaphragms [1]. Under pathological conditions, this aberrant podocyte morphology is paralleled by the severely perturbed organization of the actin cytoskeleton [2]. Thus, the actin cytoskeleton together with associated adhesion sites to the glomerular basement membrane builds the basis for the dynamic podocyte cytoarchitecture and plays a key role for proper podocyte function.

Members of the Rho family of small GTPases RhoA, Rac1, and Cdc42 have emerged as key regulators of the actin cytoskeleton [3] modulating cellular morphology, adhesion and migration [4]–[6]. Particularly, RhoA, via the downstream pathway Rho-associated protein kinase (ROCK) and myosin light chain (MLC), is an important regulator of cellular contractility and cell adhesion via generation of actin stress fibers and focal adhesions [7]–[9]. Although the actin cytoskeleton is central to podocyte function, the role of Rho GTPases in this cellular system and their regulation by upstream pathways has not been studied in much detail [10], [11].

The ubiquitously expressed serine-threonine kinase mTOR (mammalian target of rapamycin) is the catalytical subunit of two protein complexes controlling multiple aspects of podocyte function such as cell size, shape and proliferation [12]. mTOR inhibitors in general exert their actions via two functional complexes, mTOR complex 1 (mTORC1) and mTOR complex 2 (mTORC2). Whereas mTORC1 consists of mTOR, raptor, and mLST8 and regulates cell growth through effectors such as p70S6K and 4E-BP1, mTORC2 contains mTOR, rictor, and mLST8 and regulates the prosurvival kinase Akt by phosphorylating it on S473 [13], [14].

In HEK293 cells mTORC2 was linked to Rho GTPase signaling in the context of cytoskeletal regulation [15]. Activity of Rac1 GTPase was decreased in cells depleted of individual mTORC2 components and overexpression of activated Rac1 mutant led to recovery of membrane ruffles specific for this GTPase. Similarly, overexpression of constitutively active RhoA in mTORC2 depleted cells massively enhanced stress fiber generation. Despite the accumulating data from these studies, direct evidence relating mTOR signaling to Rho GTPases in podocytes is lacking [16].

Interestingly, both podocyte specific depletion as well as overactivation of the mTOR complexes in mice led to proteinuria and glomerular dysfunction suggesting that the balanced regulation of mTOR activity and the tight control of its effectors are crucial for physiological podocyte behavior [12], [17]. Today, pharmacological inhibitors of mTOR are widely-used as immunosuppressants in renal transplantation [18]. However, molecular details with respect to this tight balance and the affected downstream signaling pathways are still not well understood. Besides their immunosuppressive actions, non-immunological effects of mTOR inhibitors on the podocyte actin cytoskeleton have been described recently [16]. Prolonged treatment of cultured human podocytes with the mTORC1 inhibitor rapamycin was reported to result in alterations of the actin cytoskeleton [16]. As such extended application of rapamycin leads to unspecific inhibition of mTORC2 as well, the damage on the actin cytoskeleton might be the result of multiple affected effector activities. Consistent with this experimental data, mTOR inhibitor treatment of human renal allografts is frequently associated with the development of de novo proteinuria [19]–[21] with its incidence depending on the clinical setting [22]–[26]. Post-transplant proteinuria is a well-known phenomenon in kidney transplantation and regarded as a prognostic factor for graft and patient survival [27].

In contrast to the unfavorable effects in transplant kidneys and human podocytes, protective effects of mTOR inhibitors were demonstrated by Daniel et al. in rats in an experimental setting of puromycin aminonucleoside (PAN) induced proteinuria [28]. In this rat model, onset of proteinuria was associated with perturbed energy metabolism and alterations of the ultrastructural architecture of the podocyte. In order to inhibit mTOR signaling, Daniel et al. used everolimus (EV), a second generation inhibitor targeting both, mTORC1 and mTORC2 [15], [29]. Treatment with EV ameliorated all of the observed defects with complete remission of proteinuria indicating a possible relevance of mTOR inhibition in the therapy of proteinuric disease. Given the complexity of intracellular mTOR signaling and the need for the development of novel antiproteinuric therapeutic strategies, the delineation of mTOR related effects on the actin cytoskeleton seems highly important.

In the present study, we investigated the cellular effects of mTOR inhibition via EV and assessed the RhoA signaling pathway as a potential downstream mediator of its direct actions with respect to cytoskeleton stabilization in the PAN experimental model of proteinuric disease. We provide direct evidence that PAN-induced inactivation of RhoA in human cultured podocytes is associated with aberrant remodeling of the actin cytoskeleton and consecutive podocyte dysfunction. EV ameliorated these effects by preventing the inhibition of RhoA signaling involving ROCK and MLC. These observations might prompt further studies of mTOR inhibitors in proteinuric disease.

## Materials and Methods

### Cell Culture

Conditionally immortalized human podocytes were generated by Dr. Moin A. Saleem (University of Bristol, South Mead Hospital, Bristol, UK) [28]. Culture conditions were described previously [30].

### Experimental Design and Drug Treatment

In order to examine the non-immunological effects of EV on PAN induced cytoskeletal defects, podocytes were grown under growth restrictive conditions for 12 d and subsequently incubated with media containing 10% FBS in the presence of 30 µg/ml PAN (Sigma, Munich, Germany), 100 nM EV (Fluka, Buchs, Switzerland) or the combination of both for 48 h. All experiments were performed at least three times starting on growth-restricted days 12–14. As EV was dissolved in methanol (MeOH) appropriate amounts of the solvent was added to each control sample.

In order to further study the role of RhoA signaling in the protective effects of EV on the cytoskeleton, an inhibitor of ROCK (Y-27632, 10 µM for 1 h, Calbiochem, Darmstadt, Germany), was used in combination with PAN or PAN+EV. The inhibitor was added to podocytes after 47 h treatment.

### Apoptosis Detection

Hoechst 33342-staining of podocytes was performed as previously described [31]. Images were obtained by a Nikon ECLIPSE T_i_ Fluorescence microscope and NIS Elements AR 3.2 software (Nikon, Düsseldorf, Germany). Apoptosis was defined as percentage of cells with nuclear fragmentation. For each sample in a given experiment, at least 300 randomly chosen cells were analyzed.

### Immunofluorescence and Cell Imaging

For immunofluorescence, podocytes were plated on glass coverslips. After treatment, cells were fixed with 4% formaldehyde (Fischar, Saarbrücken, Germany) for 15 min at 37°C, washed with PBS and permeabilized with PBS/0.5% Triton X-100/3% BSA for 45 min at room temperature. After washing with blocking buffer (PBS/0.5% BSA), cells were incubated with the appropriate antibody dilutions. For paxillin staining cells were incubated with a mouse anti-paxillin antibody at 4°C over night (1∶500, BD Biosciences, San Jose, California) followed by the corresponding Alexa Fluor 488 chicken anti-mouse IgG (H+L) (1∶1000, Invitrogen, Karlsruhe, Germany) together with phalloidin-TRITC (1∶1000, Sigma, München, Germany) for actin staining in blocking buffer for 1 h at room temperature. In parallel, nuclei were stained with 4.6-diamidino-2-phenylindole dihydrochloride (DAPI, Sigma, München, Germany).

Fluorescence and brightfield imaging was performed on a fully automated Nikon ECLIPSE T_i_ microscope controlled by NIS Elements AR 3.2 software (Nikon, Düsseldorf, Germany) equipped with a CoolSNAP HQ2 camera (Photometrics, Tucson, Arizona). Images were acquired using 4× and 20× phase contrast objectives with appropriate filter sets. Time-lapse imaging was carried out in a sealed chamber at 37°C and with indicated frame rates. Image processing and analysis was performed with ImageJ (http://rsbweb.nih.gov/ij/index.html) and Adobe Photoshop software (Adobe Systems, Mountain View, CA). All images were acquired at random positions.

Confocal images were acquired on a Leica TCS SP5 confocal microscope through a 63×1.4 NA oil objective and the appropriate laser settings (Leica Microsystems, Wetzlar, Germany).

### Cell Adhesion Assay

After 48 h of pharmacological treatment human podocytes were detached using Trypsin-EDTA (Biochrom, Berlin, Germany) and seeded on glass-coverslips in a 24-well plate (1×10^5^ cells per well) for adhesion tests. After 6 h, cells were fixed with 4% formaldehyde (Fischar, Saarbrücken, Germany) and staining of the actin cytoskeleton was carried out as described above. For each condition, 25 randomly chosen regions were imaged and cell number and degree of spreading were measured using region measurement tools in ImageJ software (http://rsbweb.nih.gov/ij/index.html).

### Wound-healing Cell Migration Assay

For monitoring cell migration of PAN and PAN+EV treated podocytes, the Radius™ 24-Well Cell Migration Assay (Cell Biolabs, San Diego, USA) was used. Pretreatment of the Radius™ Migration Plate, cell seeding and Radius™ Gel Removal was performed according to manufacturer’s instructions. After a final washing step with complete medium, PAN and EV were directly added into the wells. Phase-contrast images were captured before migration and after 12 h allowing cells to migrate at 37°C in a CO_2_-incubator using a 4×objective. Wound closure was quantified by using ImageJ software (http://rsbweb.nih.gov/ij/index.html).

### Western Blot Analysis

Cells were harvested using CelLytic MT-buffer (Sigma-Aldrich, Hamburg, Germany) according to manufacturer’s instructions. Lysis-buffer was supplemented with Protease Inhibitor Cocktail (Sigma-Aldrich, Hamburg, Germany), 10 µg/ml aprotinin (Roche, Mannheim, Germany), 10 µg/ml leupeptin (Roche, Mannheim, Germany) and 2 mM phenylmethanesulfonylfluoride (PMSF, Sigma, Munich, Germany). Isolation was performed at 4°C. Total protein content was measured by Bio-Rad protein assay (Bio.Rad, Munich, Germany). Samples (each 10 µg protein) were supplemented with Laemmli sample buffer (Bio-Rad, Munich, Germany) and boiled for 10 min at 95°C. Proteins were separated using 10% or 12% Mini-PROTEAN TGX Precast Gels (Bio-Rad, Munich, Germany) and transferred on 0.45 µm PVDF Transfer Membranes (Thermo Scientific, Schwerte, Germany) with a MiniProtean Tetra Cell electrophoresis system (Bio-Rad, Munich, Germany) and a Biometra fastblot B34 blotting device (Biometra, Göttingen, Germany). 10 µl Precision Plus Protein All Blue Standard (Bio-Rad, Munich, Germany) was used as marker. Membranes were incubated with primary antibodies against RhoA (1∶500; Cell Biolabs, San Diego, USA), MLC (1∶1000; Sigma, Munich, Germany), phospho-MLC (1∶500; directed against Ser19; Cell Signaling, Danvers, USA), Akt (1∶1000; Cell Signaling, Danvers, USA), phospho-Akt (1∶1000; directed against Ser473; Cell Signaling, Danvers, USA), p70S6K (1∶1000; Cell Signaling, Danvers, USA); phospho-p70S6K (1∶1000; directed against Thr389; Cell Signaling, Danvers, USA), GAPDH (1∶10000; Sigma, Munich, Germany) or alpha-Tubulin (1∶10000; Sigma, Munich, Germany). Secondary antibodies used were horseradish peroxidase-conjugated goat anti-rabbit IgG (Santa Cruz, Heidelberg, Germany; 1∶10000 against phospho-MLC, Akt, phospho-Akt; p70S6K and phospho-p70S6K) and goat anti-mouse IgG (Santa Cruz, Heidelberg, Germany; 1∶20000 against GAPDH and 1∶10000 against MLC and RhoA). Signal detection was performed with SuperSignal West Femto Chemiluminescent Substrate (Thermo Scientific, Schwerte, Germany) and visualized by the FUSION FX7 chemiluminescence-system (PEQLAB, Erlangen, Germany) und Fusion-software (PEQLAB, Erlangen, Germany). Intensity of signals was determined using ImageJ software (http://rsbweb.nih.gov/ij/index.html). Densitometric data were normalized to alpha-Tubulin or GAPDH-loading control.

### RhoA GTPase Activation Assay

Levels of active GTP-bound RhoA were determined using a Rho GTPase Activation Assay Kit (Cell Biolabs, San Diego, USA), following the standard protocol as recommended by the manufacturer. Culture media was aspirated and cells were washed three times with ice-cold PBS. 200 µl of ice-cold assay/lysis buffer supplemented with 10 µg/ml aprotinin (Roche, Mannheim, Germany), 10 µg/ml leupeptin (Roche, Mannheim, Germany) and 1 mM PMSF (Sigma, Munich, Germany) was added to the cells and placed on ice for 20 min. Lysate cells were collected, cleared by centrifugation for 10 min (14.000 *g* at 4°C) and placed on ice. Aliquots of 0.5–1.0 ml (500 µg protein) of cell lysates were adjusted to 1 ml with assay/lysis buffer. To each sample 40 µl of Rhotekin RBD coupled Agarose bead slurry was added. Samples were incubated at 4°C for 1 h with gentle agitation. Beads were pelleted by centrifugation for 10 sec at 14.000 *g*. The supernatant was discarded, and the beads were washed three times with 0.5 ml of assay/lysis buffer. After the last wash beads were resuspended in 40 µl of Laemmli sample buffer (Bio-Rad, Munich, Germany). Each sample was boiled for 10 min and centrifuged for 10 sec at 14.000 *g*. 10 µg of total cell lysates (total RhoA) and 35 µl of the samples were analyzed by western blotting using RhoA antibody as described above. After protein-blotting an additional Ponceau S-staining (0.1% (w/v) Ponceau S in 5% (v/v) acetic acid) of the PVDF-membrane was performed to analyze the amount of Rhotekin RBD in each protein-sample. For analyses the signal of active RhoA in each sample was normalized to the corresponding total RhoA.

### Statistical Analysis

Values from multiple experiments were expressed as means ± SD. Statistical analysis was assessed using Student’s T-Test. Statistical significance was defined as *P<0.05.

## Results

### Stress Fiber Organization in Human Podocytes

**Figure 1:**
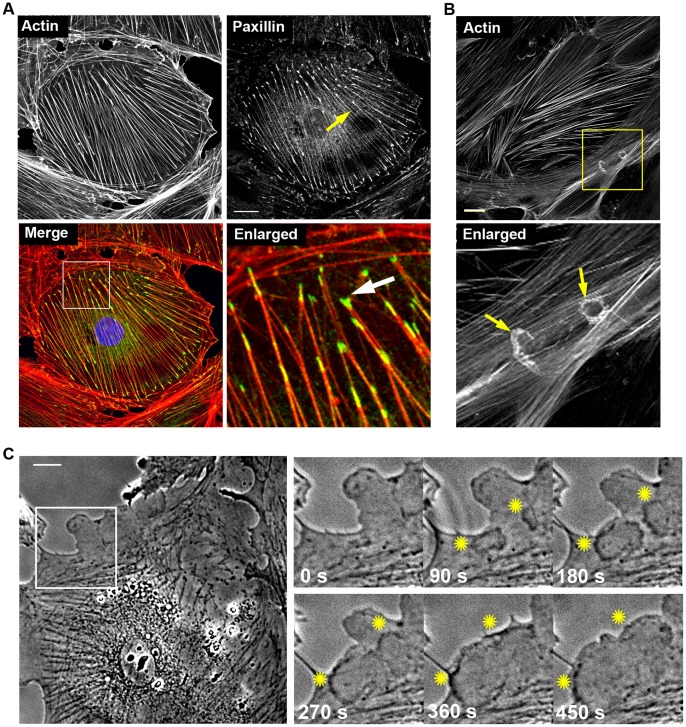
Cytoskeletal organization in differentiated human podocytes. (A) Confocal imaging of the actin cytoskeleton and focal adhesions. Actin was visualized by phalloidin-TRITC and focal adhesions by paxillin antibody staining. Actin and paxillin images are presented in gray scale to preserve maximum contrast. Merge image shows actin in red and paxillin in green. Yellow arrow in the paxillin image: Weak paxillin staining along stress fibers. White arrow in the enlarged image: Strong accumulation of paxillin in focal adhesions at the ends of stress fibers. Scale bar = 20 µm. (B) Confocal imaging of the actin cytoskeleton (phalloidin-TRITC, grey) revealed distinct cellular structures reminiscent of dynamic actin rich protrusions (yellow arrows). Scale bar = 25 µm. (C) Phase contrast movies of control podocytes confirmed several dynamic protrusions generated in more central regions in addition to the peripheral extensions. Upper image depicts the first frame of a representative phase contrast movie (Movie S1). Lover panel shows the image sequence of the enlarged region (white box) with two dynamic protrusions (yellow stars).

Prior to pharmacological treatments, we investigated the cellular morphology and cytoskeletal organization of cultured human podocytes in detail. Differentiated podocytes typically display a rather non-polarized phenotype in 2D with peripheral cell-cell contacts forming the basis for the functional glomerular filtration barrier in vivo. Confocal microscopy of the actin cytoskeleton in untreated individual podocytes revealed a dense meshwork of linear actin stress fibers in the cell center spanning the entire cell (Fig. 1A, actin image). In addition, we observed distinct actin rich regions in central areas of the cell body reminiscent of small dynamic protrusions (Fig. 1B). Indeed, in phase contrast live-cell experiments we were able to detect multiple transient protrusions in individual cells revealing the highly dynamic behavior of the cytoskeleton in differentiated podocytes (Fig. 1C, Movie S1).

In addition to the actin structures, focal adhesions were visualized using antibody staining against the adaptor protein paxillin. As expected, focal adhesions were associated with the ends of stress fibers connecting the cytoskeleton with the substrate (Fig. 1A, enlarged, white arrow). However, in contrast to other cell types such as fibroblasts, focal adhesion localization was not strictly restricted to these distal regions but was also detected along the entire length of stress fibers (Fig. 1A, paxillin image, yellow arrow).

### EV Prevents Disruption of the Actin Cytoskeleton

**Figure 2:**
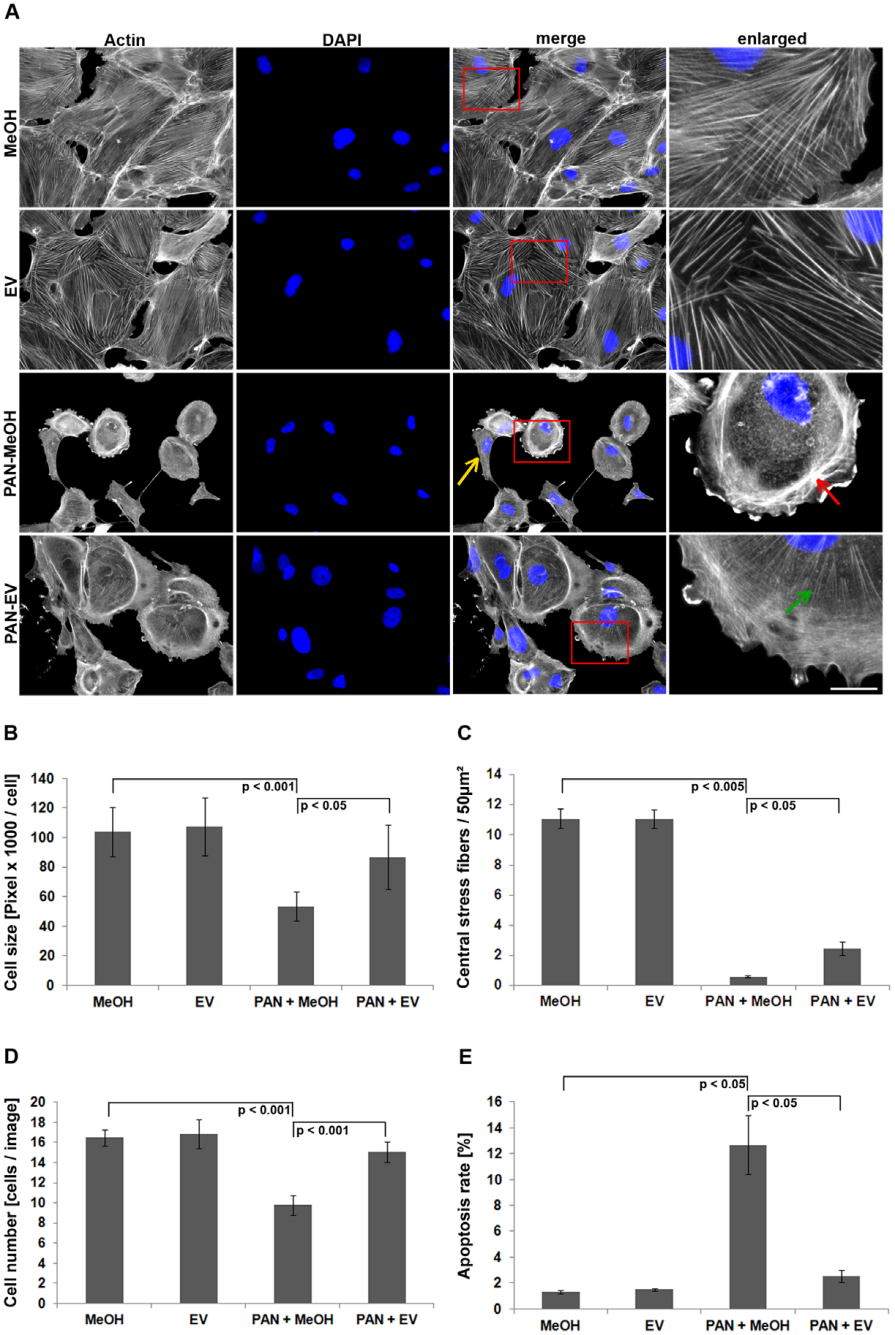
EV prevents disruption of the actin cytoskeleton in human podocytes. (A) Actin (phalloidin-TRITC, grey) and nuclear staining (DAPI, blue). Scale bar = 100 µm. (B) Quantification of cell size (n = 5 experiments, ≥25 images per condition). (C) Number of central actin stress fibers within a distinct area (50 µm^2^) (n = 3 experiments, 20 cells per condition). (D) Quantification of cell numbers (n = 5 experiments, ≥25 images per condition). (E) Hoechst nuclear staining for the detection of apoptosis (n = 3 experiments, ≥50 images per condition). Apoptotic cells were defined as percentage of fragmented nuclei. MeOH = solvent for EV. Data are means ± SD.

Studies of human podocytes indicated direct effects of mTOR inhibitors on the podocyte cytoskeleton besides their previously suggested immunosuppressive actions. In proteinuric animal models, the application of the mTOR inhibitor EV was demonstrated to significantly reduce proteinuria [32]. To study the molecular effects of EV in podocyte injury in detail, we applied puromycin aminonucleoside (PAN) as a well-recognized in vitro model. As expected, treatment with PAN (30 µg/ml for 48 h) caused strong morphological and cytoskeletal defects. Overall, treated cells were significantly smaller and often adopted a front-to-back polarized shape reminiscent of migratory fibroblasts (Fig. 2A and B, yellow arrow). More prominently, central stress fibers were diminished significantly after exposure to PAN (Fig. 2C). Instead, we noticed substantial accumulation of thin and less organized actin fibers at the cell periphery (Fig. 2A, red arrow).

When exposed to EV (100 nM) along with PAN (PAN+EV), cell body size enlarged (Fig. 2B) and number of podocytes with central stress fibers increased significantly (Fig. 2A, green arrow; and C). In addition, cells displayed a less aberrant organization of stress fibers as compared to PAN alone (Fig. 2C). Interestingly, EV alone did not affect podocyte morphology or the actin cytoskeleton suggesting that the compound might specifically act on signaling pathways altered in podocyte damage (Fig. 2A and C).

As we observed substantial podocyte loss following PAN treatment (Fig. 2D), we subsequently tested whether this massive decrease in cell numbers was due to apoptosis. DNA fragmentation was quantified by Hoechst-staining in human podocytes after exposure to PAN for 48 h. In agreement with recent data [31] PAN treatment led to a significant induction of apoptosis as the number of cells with fragmented DNA increased dramatically (Fig. 2E). Combining PAN treatment with EV led to significant increase in cell number paralleled by reduced apoptosis as compared to PAN treatment alone (Fig. 2D and E), approximating the basal apoptosis rate of control cells. These results confirm that in addition to protective effects on the cytoskeletal organization EV is an antiapoptotic factor for podocytes in response to PAN and thereby maintains podocyte viability.

### EV Reduces Podocyte Motility

**Figure 3:**
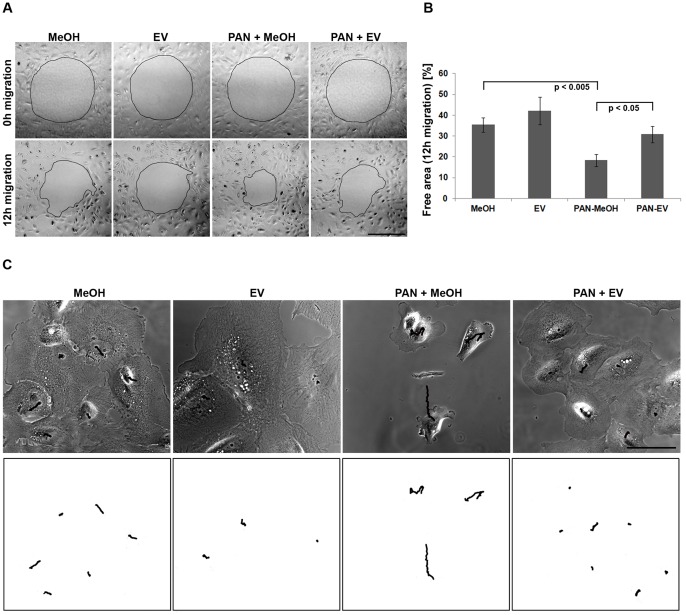
EV inhibits migration in human podocytes. (A) Representative phase contrast images after 0 h and 12 h of migration. Scale bar = 500 µm. (B) Quantification of migration efficiency by measurement of cell-free area after 12 h of migration (n = 3 experiments, ≥5 images per condition). (C) Phase contrast time-lapse studies of living cells. Migration was tracked following the nuclei in the phase contrast movie. Lower panel: Tracks were depicted on white background for better contrast. Scale bar = 500 µm. MeOH = solvent for EV. Data are means ± SD.

Proper organization and stability of central actin stress fibers and the associated cell-substrate adhesions are essential for podocyte anchorage to the basement membrane. In contrast, loss of the central actin bundles and a pronounced front-to-back polarization, as we observed after PAN treatment, are elementary for the movement of migratory cells. In agreement with our observation, a previous study reported increased migration behavior of murine podocytes after PAN treatment [33]. Similar to the effect in murine cells, PAN treated human podocytes migrated faster in an in vitro scratch assay as compared to control cells (Fig. 3A and B). The addition of EV substantially decreased migration efficiency to a level similar to control cells. Consistent with this finding, higher magnification time-lapse imaging of single podocytes and tracking of movement revealed that addition of EV leads to a substantial loss of PAN induced dynamics and adaptation of a more stationary podocyte phenotype (Fig. 3C). Overall, EV treatment alone did not affect migration behavior.

### EV Enhances Podocyte Adhesion

**Figure 4:**
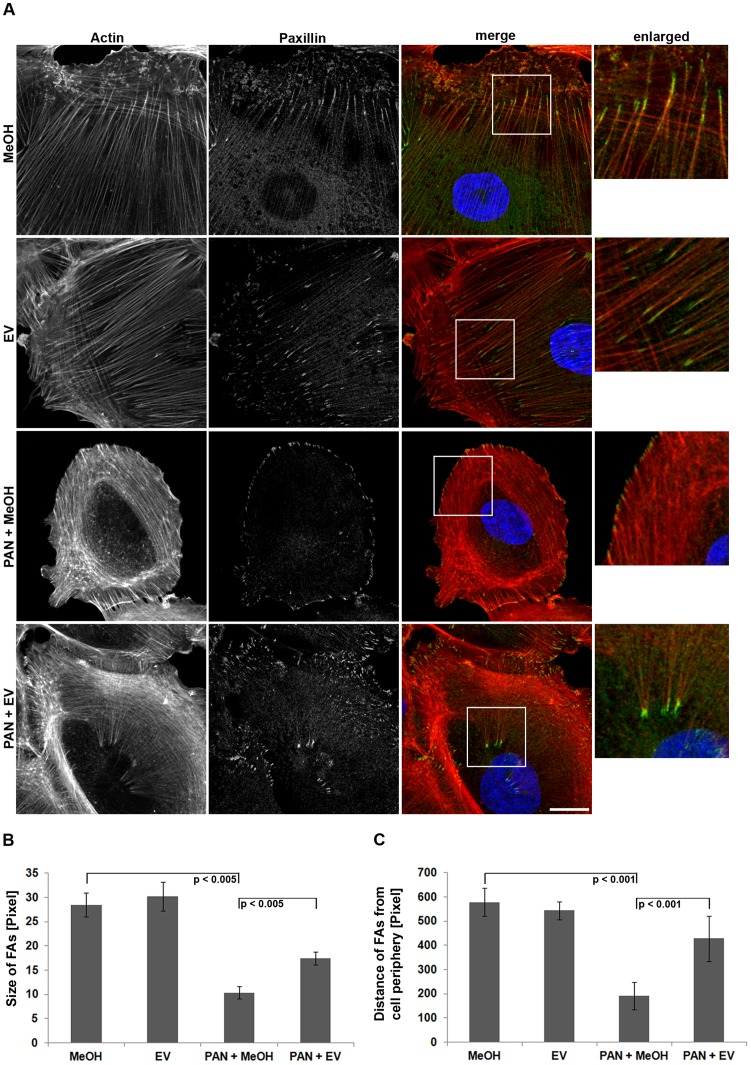
Aberrant distribution and size of focal adhesions is recovered by EV in human podocytes. (A) Actin (phalloidin-TRITC) and paxillin (antibody staining) images are presented in gray scale for maximum contrast. The merge image depicts paxillin in green and actin in red. DAPI was used to visualize nuclei (blue). White arrows depict focal adhesion localization. Scale bar = 25 µm. (B) Quantification of focal adhesion size (n = 3 experiments, ≥10 images per condition). (C) Quantification of the distance of focal adhesions from the cell periphery (n = 3 experiments, ≥10 images per condition. MeOH = solvent for EV. FAs = focal adhesions. Data are means ± SD.

The actin cytoskeleton is associated with the glomerular basement membrane via integrin triggered focal adhesions. Critically, the maturation and turn-over of focal adhesions is intimately linked to actin-myosin contractility [34]. Thus, given the aberrant organization of the actin cytoskeleton during PAN induced injury we carefully assessed the size and spatial organization of focal adhesions by paxillin staining. Overall, a significant decrease in the average length of focal adhesions was revealed when cells were treated with PAN (Fig. 4A and B). In addition, we also found that paxillin was localized at close proximity to the cell edge, whereas adhesions in control cells were found in substantial distance from the cell periphery (Fig. 4C). In parallel to the recovery of the actin cytoskeleton, the size of focal adhesions and the distance from the edge was substantially increased when cells were incubated with PAN+EV (Fig. 4A and B).

**Figure 5:**
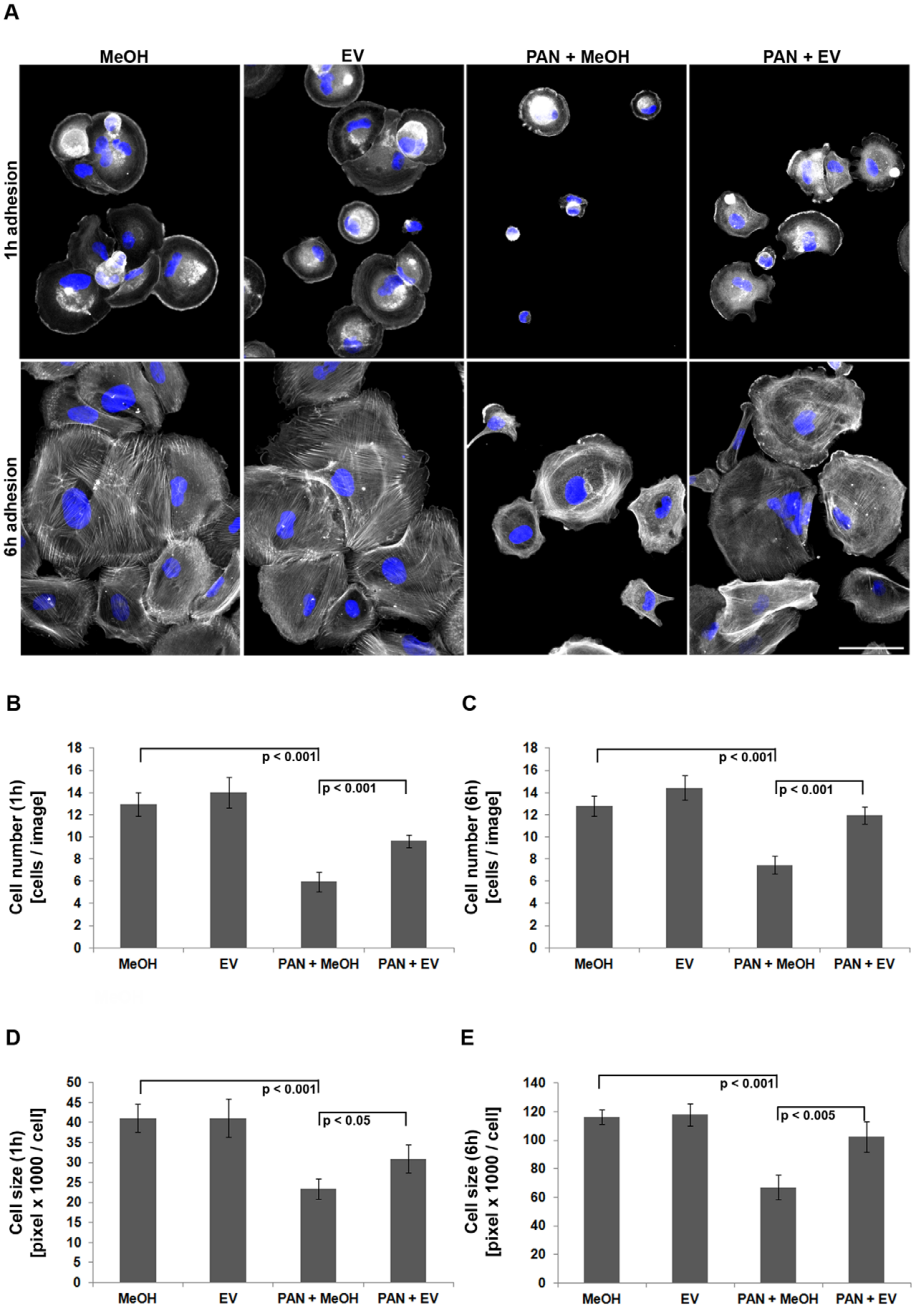
Decreased podocyte adhesion due to injury is partially recovered by EV. (A) Actin (phalloidin-TRITC, grey) and nuclear (DAPI, blue) staining at 1 h and 6 h after post-plating. Scale bar = 100 µm. (B and C) Quantification of cell numbers after 1 h and 6 h post plating (n = 4 experiments, ≥25 images per condition). (D and E) Quantification of cell size after 1 h and 6 h post plating (n = 4 experiments, ≥25 images per condition). MeOH = solvent for EV. Data are means ± SD.

The decrease in focal adhesion size together with the observed reduction in cell body size following PAN treatment suggested defects in cellular adhesion and spreading. Thus, we quantified adhesion efficiency by measuring cell number and size of podocytes at 1 h and 6 h post-plating on glass coverslips. PAN treated podocytes adhered and spread less efficiently as compared to control cells (Fig. 5A–E). However, when cells were treated with PAN+EV, the lack of adhesion efficiency and decrease in spreading area was recovered substantially.

As depicted in Fig. 5A, fluorescence imaging of the actin cytoskeleton in replated podocytes revealed severe perturbation of central stress fibers in PAN treated cells, indicating that PAN not only interferes with the long-term maintenance (stability) but also the de novo generation of stress fibers, both processes regulated by RhoA signaling.

### EV Inhibits mTORC1 and mTORC2

**Figure 6:**
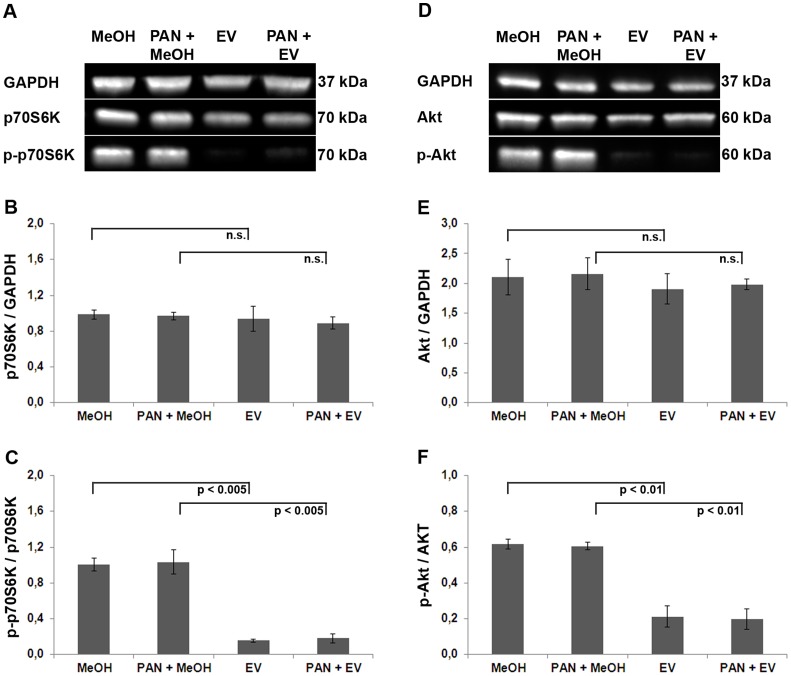
EV inhibits mTORC1 and mTORC2. (A) Western blot analysis to measure the activation level of Akt (representative example from 3 independent experiments). GAPDH = loading control, Akt = total Akt protein levels, pAkt = active, phosphorylated Akt protein. (B) For quantification, total Akt protein was normalized with respect to GAPDH. (C) To quantify the amount of active Akt protein, phosphorylated Akt was normalized with respect to total Akt. (D) Western blot analysis of p70S6K protein (representative example from 3 independent experiments). GAPDH = loading control, p70S6K = total p70S6K protein levels, p-p70S6K = active, phosphorylated p70S6K protein. (E) For quantification, total p70S6K was normalized to GAPDH. (F) Phosphorylated p70S6K was normalized to total p70S6K.

In mTORC1 mTOR is known to phosphorylate Akt whereas in mTORC2 p70S6K is phosphorylated by the kinase [13], [14], [35]. In order to confirm inhibition of either mTOR complex by EV treatment in our experimental setting, we analyzed the phosphorylation status of Akt and p70S6K (Fig. 6A–F). EV substantially decreased phosphorylation level of both mTOR effector proteins (Fig. 6A–F). Based on these data, we suggest that EV is a potent inhibitor of both mTORC1 and mTORC2 in human podocytes.

### EV Targets RhoA Signaling Pathway

**Figure 7:**
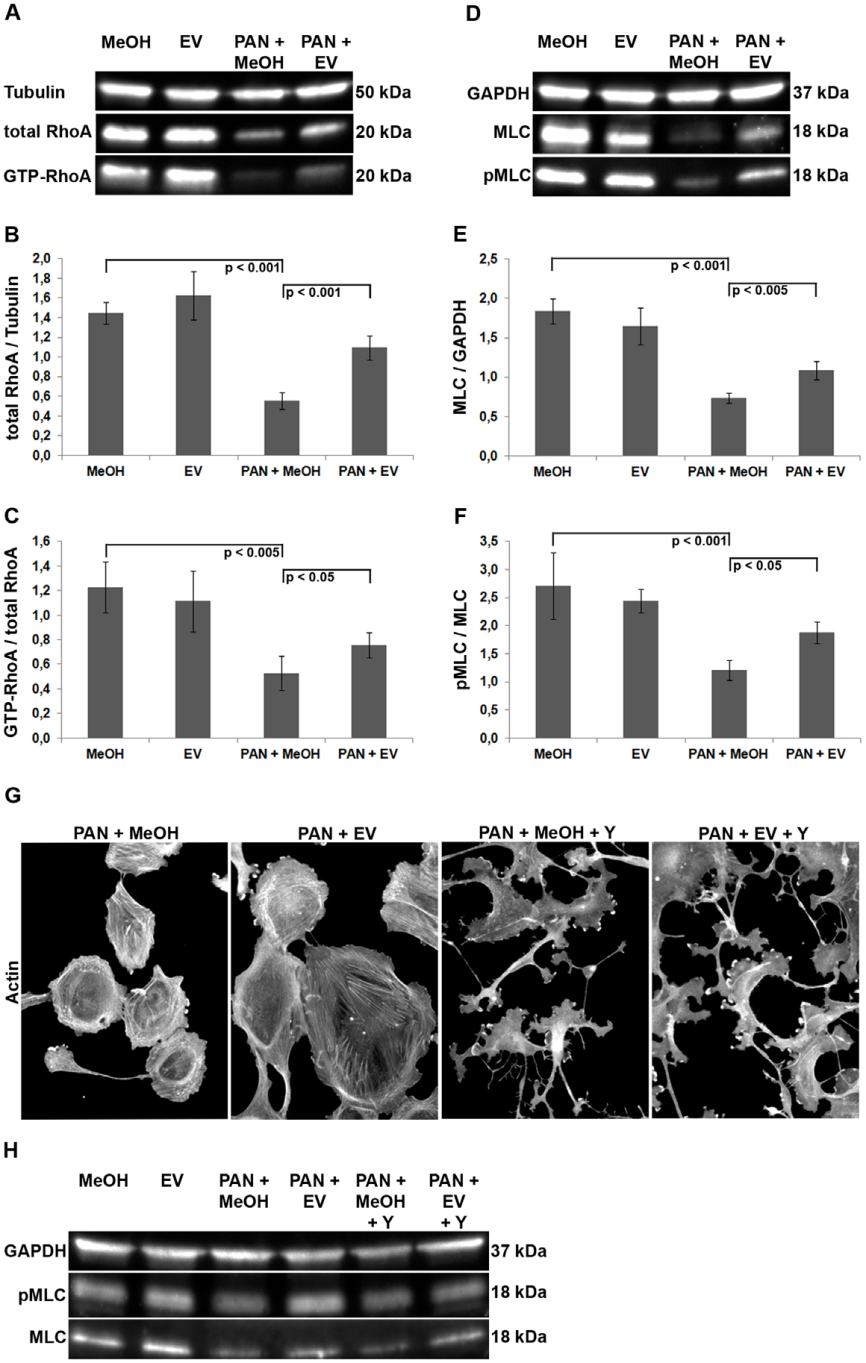
EV targets RhoA signaling pathway in human podocytes. (A) Biochemical assay to measure the activation level of the GTPase. The Rho-binding domain (RBD) of the RhoA effector rhotekin was used to affinity-precipitate the active fraction of endogenous RhoA (GTP-RhoA) from cell lysates (representative example from 3 independent experiments). Tubulin was used as loading control. (B) Quantification of total RhoA protein (n = 3 experiments). For quantification, total RhoA protein was normalized with respect to tubulin from whole cell lysates. (C) To quantify the amount of active RhoA protein, GTP-bound RhoA was normalized with respect to total RhoA (n = 3 experiments). (D) Western-blot analysis of MLC protein (representative example from 4 independent experiments). GAPDH = loading control, MLC = total MLC protein levels, pMLC = active, phosphorylated MLC protein. (E) Quantification of total MLC protein (n = 4 experiments). For quantification, total MLC was normalized to GAPDH from whole cell lysates. (F) Quantification of phosphorylated MLC protein (n = 4 experiments). Phosphorylated MLC was normalized to total MLC from whole cell lysates. (G) Western blot analysis of MLC protein after treatment with the ROCK inhibitor Y-27632 (10 µM for 1 h; n = 2 independent experiments). (H) Actin cytoskeleton (phalloidin-TRITC, grey) after treatment with Y-27632. DAPI was used for nuclear staining (blue). Scale bar = 100 µm. MeOH = solvent for EV. Data are means ± SD.

The Rho GTPase RhoA is a critical regulator of cellular contractility and focal adhesion dynamics [36], [37]. The significant recovery of the central actin bundles in the presence of EV suggested that signaling pathways related to the stress fiber regulating protein RhoA might be restored in the presence of EV. Thus, we first measured protein as well as activity level of RhoA after PAN treatment and in the presence of EV. PAN dramatically reduced the amount of total RhoA protein (Fig. 7A and B). In addition, by glutathione S-transferase pull-down experiments we found a substantial reduction of RhoA activity as compared to control cells indicating aberrant upstream signaling pathways controlling the activity state of the GTPase (Fig. 7A and C). Strikingly, addition of EV restored both RhoA protein amount as well as activity (Fig. 7A, B and C). These findings suggest that EV might prevent PAN induced inhibition of RhoA signaling via recovery of pathways regulating both protein biosynthesis as well as activity of the GTPase.

The serine-threonine kinase ROCK (Rho-associated protein kinase) is known to mediate stress fiber formation downstream of RhoA via increase of myosin light chain (MLC) phosphorylation [38]. Thus, stability of stress fibers is strictly connected to persistent activity of ROCK as acute inhibition of the kinase shortly leads to the disruption of stress fibers [39]. To further substantiate the relevance of the RhoA-ROCK-MLC pathway in EV-mediated cytoskeletal recovery, MLC activity was measured by western blot analysis of MLC phosphorylation. Consistent with the effects on RhoA, protein amount and phosphorylation of MLC significantly decreased after treatment with PAN, but increased in the presence of EV (Fig. 7D, E and F).

In order to test if ROCK activity is involved in EV-mediated activation of MLC during PAN injury, podocytes were exposed to a specific inhibitor of the kinase, Y-27632. Addition of the compound to the PAN+EV treatment for 1 h diminished the protective effects of EV on central actin stress fibers entirely (Fig. 7G). Cells adopted the characteristic cellular morphology reminiscent of ROCK inhibition including the loss of central actin bundles and generation of prominent branched extensions.

Furthermore, the recovery of MLC phosphorylation mediated by EV was substantially reduced when ROCK was inhibited by Y-27632 confirming that the kinase is involved in the upregulation of MLC activity by EV (Fig. 7H).

## Discussion

Alterations of podocyte ultrastructure, in particular its foot processes, are the histologic hallmark of glomerular disease associated with severe proteinuria, and are closely linked to disaggregation and altered distribution of actin filaments [2]. The present study addresses cytoskeleton-related pathogenetic effects of the mTOR inhibitor everolimus in an in vitro model of human podocyte injury assessing the potential of mTOR inhibition for future therapy regimens of proteinuric disease.

First, we thoroughly studied the dynamics of the actin cytoskeleton and cell morphology of human podocytes. Confocal microscopy of non-treated cells revealed a dense meshwork of linear actin stress fibers spanning throughout the entire cell. In addition, distinct hemispherically shaped actin rich regions within the cell body were observed. These could be correlated to the occurrence of transient protrusions by phase contrast live-cell experiments revealing the highly dynamic behavior of the cytoskeleton in differentiated podocytes. The significance of stress fibers in cultured podocytes is still under debate. It has been shown that the actin cytoskeletal structure in the foot processes of adult rat podocytes is divided into two types of networks [40]. Whereas actin bundles run along the longitudinal axis of the foot process above the level of the slit diaphragm, the cortical actin network is distributed beneath the plasma membrane of the foot process. Podocyte foot process effacement, the hallmark of podocyte injury and proteinuric kidney disease, is often accompanied by the disappearance of these actin filaments [41]. Mundel *et al.* showed that in differentiated murine podocytes, the actin cytoskeleton was rearranged into fibroblast-like stress fibers extending into the processes [42]. It is well established that stress fibers in cultured podocytes correspond to the filamentous actin in podocyte foot processes *in vivo* and as such represent differentiation of podocytes [41].

Adhesion of podocytes to the glomerular basement membrane is mediated by integrin triggered focal adhesions associated to the actin cytoskeleton (reviewed in Kretzler, 2002) [43]. The maturation and turn-over of these adhesions is intimately linked to actin-myosin contractility [44]. Interestingly, focal adhesion distribution in podocytes was found to be different as compared to other cell types (e.g. fibroblasts). Adhesions were located along the entire length of actin stress fibers instead of being restricted to their end, an anatomical precondition for the extensive but dynamically regulated adhesion to the glomerular basement membrane.

PAN is a toxic molecule used to induce experimental proteinuria in animals [45], [41]. It alters the stability of the podocyte cytoskeleton associated with the effacement of foot processes, making it a reliable model for glomerular diseases under experimental conditions [46]. In rodents, the strong cytoskeletal damage induced by PAN has been previously shown to be rescued by different pharmaceutical agents [47], [48]. We used this in vitro approach to investigate possible rescue effects of EV. We confirmed the damaging effects of PAN treatment in our in vitro model of differentiated human podocytes. Treatment caused strong morphological and cytoskeletal defects with significant reduction of cell size, a pronounced front-to-back polarized cell shape and significant loss of central actin stress fibers, all elementary processes required for the movement of migratory cells. Consistently, we measured a substantial increase in the migration efficiency of human podocytes similar to previous reports utilizing murine cell systems [33], [49].

In addition to the loss of central actin stress fibers, we detected decreased cell adhesion after PAN treatment accompanied by significant shortening of focal adhesions and their absence in the cell body. These changes in focal adhesion size and adhesion efficiency might be directly linked to the massive defects of the actin cytoskeleton as focal adhesion formation and their turn-over have been associated with cellular tension generated by actin stress fiber contractility [50]. Interestingly, we also measured strongly enhanced apoptosis by PAN similar to a previous study in human podocytes [31]. The resulting detachment of podocytes is discussed to be a critical event in the cascade of glomerular damage in patients with proteinuric disease [51] and might be related to mechanisms of apoptosis associated with lack of adhesion (anoikis).

Addition of the mTOR inhibitor EV proved to be protective regarding the deleterious effects of PAN treatment. Particularly, PAN induced apoptosis was prevented almost entirely. In the literature, effects of EV on apoptosis are controversially discussed. Whereas some publications report no influence of EV on apoptosis or even decrease in cell survival [16], [52], Marcova et al. demonstrated, similar to our results, a protective effect of EV against pemetrexed-induced apoptosis in non-small cell lung cancer [53]. The significant decrease of apoptosis by EV in our study is paralleled by concomitant effects on podocyte morphology, adhesion and migration. EV substantially decreased PAN induced cell motility by promoting a stationary podocyte phenotype with increased cell size, circularity and cell adhesion. Particularly, EV treatment prevented partially the loss of prominent central actin stress fibers together with associated focal adhesions resulting in enhanced cell adhesion and likely averting PAN induced apoptosis. Substantial inhibition of both mTOR complexes by EV was confirmed via measurement of effector phosphorylation. Both p70S6K as well as Akt phosphorylation were decreased significantly.

Tightly balanced mTOR activity seems essential in podocyte homeostasis as both, podocyte specific depletion as well as over-activation of the mTOR complexes is associated with alterations of the podocyte ultrastructure [16], [29]. Both enhanced and decreased motility have been associated with proteinuric disease, defining a more migratory or more stationary phenotype, respectively [54]. The results of the present study confirm a hypermotile state of podocytes after PAN induced injury, rescued in part by mTOR inhibition, indicating a critical role of mTOR signaling for glomerular function. In contrast, inhibition of mTOR in healthy podocytes might affect podocyte motility differentially resulting in a hypomotile phenotype. This hypothesis seems to be supported by the observation of Vollenbröker et al. describing reduced migration of healthy human podocytes after treatment with the mTOR inhibitor rapamycin [16]. In our setting, however, application of EV alone did not affect the general podocyte morphology or the organization of the actin cytoskeleton. Given the particular stationary morphology of differentiated podocytes and the stability of their central stress fibers, this might be possibly due to dose and time course related reasons.

PAN treatment dramatically perturbed stress fiber organization indicating interference with their long-term maintenance and de novo generation, processes dependent on RhoA signaling [7], [55], [56]. As the GTPase RhoA was previously shown to be regulated by mTOR inhibition via mTORC2 [57], [58], altered RhoA signaling might be involved in the recovery of stress fibers upon EV treatment. Thus, we analyzed RhoA and its downstream effector pathway ROCK-MLC in this context [38]. PAN dramatically reduced activities of RhoA and MLC. Conclusive with the modest but significant recovery of actin stress fibers, simultaneous treatment with EV partially recovered both activities. In addition, pharmacological inhibition of ROCK with Y-27632 diminished the EV induced rescue of actin stress fibers and MLC activation confirming the relevance of the RhoA-ROCK-MLC pathway in this setting. Thus, EV might exert direct modulation of RhoA signaling leading to enhanced actin filament stability in PAN induced proteinuria.

To date, the therapy of proteinuric diseases is based on immunosuppressive agents, in particular steroids and cyclosporine A (CsA). Preceding our study, Faul et al. demonstrated that the beneficial effects of CsA on proteinuria are not only related to its immunosuppressive function but also to stabilization of the podocyte actin cytoskeleton [59]. However, as CsA exhibits considerable nephrotoxicity, other therapeutic agents with comparable effects on the podocyte cytoskeleton but lesser side effects are highly demanded.

We first describe mTOR inhibitor related effects on the podocyte actin cytoskeleton as beneficial in the context of proteinuric disease. Particularly, our results indicate a stabilizing function of EV on podocyte morphology and viability mediated by the RhoA-ROCK-MLC pathway. Further evaluation of these findings in clinical studies will help to assess whether mTOR inhibitors are to be included in future therapy regimens in order to improve the outcome of patients with proteinuric disease.

## Supporting Information

Movie S1 **Dynamic protrusions in podocytes.** Corresponding phase contrast movie to Figure 1C (lower panel) depicting the generation of two dynamic protrusions. Scale bar = 25 µm.
(AVI)
